# Urinary Dysfunction Assessment in Long-Term Survivors of Carcinoma Cervix Using LENT SOMA Scale: An Indian Study Addressing Quality of Life Issues

**DOI:** 10.31557/APJCP.2019.20.2.383

**Published:** 2019

**Authors:** Abhishek Shankar, Jaineet Patil, Niharika Sethi, Abhijit Chakraborty, Sachidanand Jee Bharati, Kavita Mandrelle, Anil Luther, Ruchir Bhandari, Goura Kishor Rath

**Affiliations:** 1 *Department of Preventive Oncology, *; 5 *Department of Oncoanaesthesia and Palliative Medicine,*; 9 *Department of Radiation Oncology, Dr B.R.Ambedkar Institute Rotary Cancer Hospital, All India Institute of Medical Sciences,*; 3 *Department of Obstetrics and Gynecology, Maulana Azad Medical College, New Delhi, *; 2 *Department of Radiation Oncology,*; 6 *Department of Obstetrics and Gynecology, *; 7 *Department of General Surgery, Christian Medical College, Ludhiana,*; 8 *Department of Radiation Oncology, Manipal Hospital, Jaipur, India, *; 4 *The Hormel Institute-University of Minnesota, Austin, MN, USA. *

**Keywords:** Cervical cancer, urinary dysfunction, quality of life (QOL)

## Abstract

**Background:**

Carcinoma cervix is the second most common type of cancer in the world. With the increasing proportion of women surviving carcinoma of the cervix, quality of life has been an important clinical issue. Since there are very few studies from India, this study is to assess urinary dysfunction issues in patients of carcinoma cervix treated with multimodality therapy using the LENT SOMA scores.

**Methods:**

The study was prospective and patients treated between 1995 - 2007 on follow up were included in this study after ethical clearance. A total of 85 patients were accrued comprising 6 stage IB, 6 stage II A, 25 stage II B, 2 stage IIIA, 45 stage III B and 1 stage IV A disease. Sixty-six patients were treated with radiotherapy in which 46 patients received chemoradiotherapy and 19 had surgery prior to post-operative radiotherapy. The mean age was 47.81 years with a range of 25-68 years. Completion of LENT SOMA scale and Statistical analysis was done.

**Results:**

Mean score for BU (Bladder/Urethra) was highest (0.0758) in fifth year of treatment whereas UK (Ureter/Kidney score was highest (0.0408) after 4 years. Bladder score was more in 60-69 years of age and in stage IIIB patients of cervical cancers. Bladder morbidity was more in patients who received chemoradiotherapy and in patients who received radiotherapy with boost where Bladder and Urethra morbidity was more in patients who were treated with Extended Field radiation.

**Conclusions:**

The LENT SOMA system was acceptable and feasible to use and gave us an insight into the morbidity in our patients and to develop effective management plans to reduce the post treatment symptoms and improve quality of life.

## Introduction

Cervical cancer is the fourth most common cancer among women worldwide, with an estimated 528,000 new cases and 266,000 deaths in the year 2012 (Ferlay 2015) and about 85% of the cases occur in developing countries (Forman et al., 2013). India, which accounts for one sixth of the world’s population, also bears one fifth of World’s burden of cervical cancer (Shankar et al., 2017A). As per ICMR 2016 report, cervical cancer incidence is 100,000 and showing a downward trend (Bobdey et al., 2016). 

 Globally 27% of total cervical cancer cases are from India which is home to 16-17% of world’s women population (Shankar et al., 2017B).

The usual treatment for patients with early stage cervical cancer is radical hysterectomy plus pelvic lymphadenectomy and concurrent chemo radiation for late stages. Radiotherapy, typically consisting of external beam radiation followed by intracavitary radiation, Treatment-related side effects include urinary, gastrointestinal, sexual, and neurological which hamper long-term quality of life. Over the past 40 years, mortality and morbidity resulting from carcinoma of the cervix has fallen because of improved treatment and the introduction of national screening programs (Shankar et al., 2018).

The outcome of Cancer treatment has been measured till now with terms of objective response rates, progression free survival and overall survival. It has become increasingly evident that impact of disease and treatment on a patient’s QOL is an important measure of effective cancer management (Rehse, 2015). Health-related quality of life can be defined as the extent to which one’s usual or expected physical, emotional, and social well-being are affected by a medical condition or its treatment (Cella, 1995). Since treatment is curative for 90% of patients with stage I cervical cancer and more than 50% women are younger than 50 years, it is important to analyse the impact on long term quality of life as they may continue to live with the sequelae of disease and its treatment. 

Bladder damage post radiotherapy is a known complication. It can manifest as urgency, frequency, nocturia and urge incontinence. Storage symptoms like decreased bladder capacity and compliance are common than voiding problems (Parkin et al. 1987). Such urinary dysfunction can cause psychological and social distress to females in form of anxiety, irritability and low self-esteem; and all this after being treated of their malignancy. Late Effects Normal Tissues(LENT)-Subjective, Objective, Management, Analytic (SOMA) (Davidson, 2002) is a scale designed to measure and record the late effects of radiotherapy to extrapolate mainly objective morbidity of malignancies and provide additional information on subjective morbidity.

Our main objective was to assess the urinary dysfunction as an important Quality of Life indicator in treated cases of carcinoma cervix by using the LENT SOMA scores. 

## Materials and Methods

*Patients*

Ethical approval was obtained from college Ethics Committee. The study was conducted in Department of Radiotherapy of Christian Medical College, India from 1st September 2009 to 31st August 2010 in patients with histologically confirmed carcinoma of uterine cervix treated between 1st January 1995 to 31st December 2007 by various modalities such as surgery, radiotherapy and chemotherapy. The inclusion criteria for the study was: (A) Patient must be a histologically proven case of carcinoma uterine cervix and (B) Patient must be treated with surgery, radiotherapy, chemotherapy alone or in combination.

Pretreatment evaluation was done which included a detailed history and general history and examination, systemic and local (Including bimanual pelvic and rectal examination), KPS, Height, Weight, Body surface area, Histopathology, Complete haemogram, Blood urea, Serum creatinine, Chest x-ray, Intravenous pyelogram, Cardiology clearance for chemotherapy, Cystoscopy, USG Abdomen and pelvis in bulky disease, Urine and stool examination if needed. All patients are staged according to FIGO staging (The American Cancer Society). 

In this study, patients included were in age range of 25-68 years with a mean age of 47.81 years. Age distribution of patients showed 23.5 % were aged less than 40 years, 28.2% fell in age group 40-49, 30.6% in age group 50-59 and 17.64% in age group 60-69 years. Thus majority of patients were noted in age group 40-59 years accounting for 58.81%. 

A total of 85 patients were accrued comprising 6 stage IB, 6 stage II A,25 stage II B, 2 stage IIIA, 45 stage III B and 1 stage IV A disease. A retrospective analysis was conducted by going through the records of these patients. Sixty-six patients were treated with radiotherapy in which 46 patients received chemo radiotherapy and 19 had surgery prior to post-operative radiotherapy. 

Radiotherapy was given according to the Manchester school (Hunter, 1991). Early stage patients were treated with radical hysterectomy followed by postoperative external beam radiotherapy and vault intra-cavitary treatment. Patients treated with radiotherapy alone received external beam irradiation with a four-field technique with no midline shielding (50 Gy over 5 weeks in 25 fractions) followed by a single intracavitary insertion giving an A point dose of 22.5–37.5 Gy. 

*Completion of the LENT SOMA scale on Urological dysfunction*

Data were collected prospectively using a questionnaire derived directly from the published LENT SOMA scales during a personal interview with the patients. However, it should be noted that the LENT SOMA scales were used as published and that the questionnaires just provided a means of obtaining the data to complete the scales.

In the published scales each answer is scored from 0 to 4 depending on the severity of the symptoms, the higher the score the more severe the symptom. The final score was recorded in two ways: as the highest or maximum for urinary dysfunction scales and as an average score for each scale. If more than 50% of the questions were not answered, then the average score was defined as missing. The score was converted to a mean by dividing by the number of questions answered. Patients were assessed on the day of follow up in Radiotherapy Out Patient Department during the study period. 

The information obtained in the questionnaires was transferred to a computer database and, as stated above, only the items listed in the published LENT SOMA system were scored. Although subjective, objective and management data were obtained. Patients were withdrawn from the study in the event of tumor recurrence/progression.

*Statistical analysis*

Data were entered onto a computer database and analyzed using SPSS (Statistical Package for Social Sciences) version 9.0. The score was converted to a mean by dividing the number of items completed. If more than half the items were missing, then the score was regarded as missing. The mean LENT subjective urinary dysfunction subscale scores were not normally distributed and so non-parametric tests were used. 

## Results

*Analysis*

A total of 735 diagnosed cases of carcinoma uterine cervix were treated by various treatment modalities alone or in combination in Department of Radiotherapy, Christian Medical College, and Ludhiana from between 1st January 1995 to 31st December 2007). This study was conducted in the Department of Radiotherapy, Christian Medical College, and Ludhiana from 1st September 2009 to 31st August 2010 on 85 diagnosed and treated patients (Between 1st January 1995 to 31st December 2007) of carcinoma uterine cervix who visited Radiotherapy OPD during study period. Statistical analysis was performed on a total of 85 patients who were interviewed for late effects of treatment using LENT- SOMA scale. 

**Figure 1:**
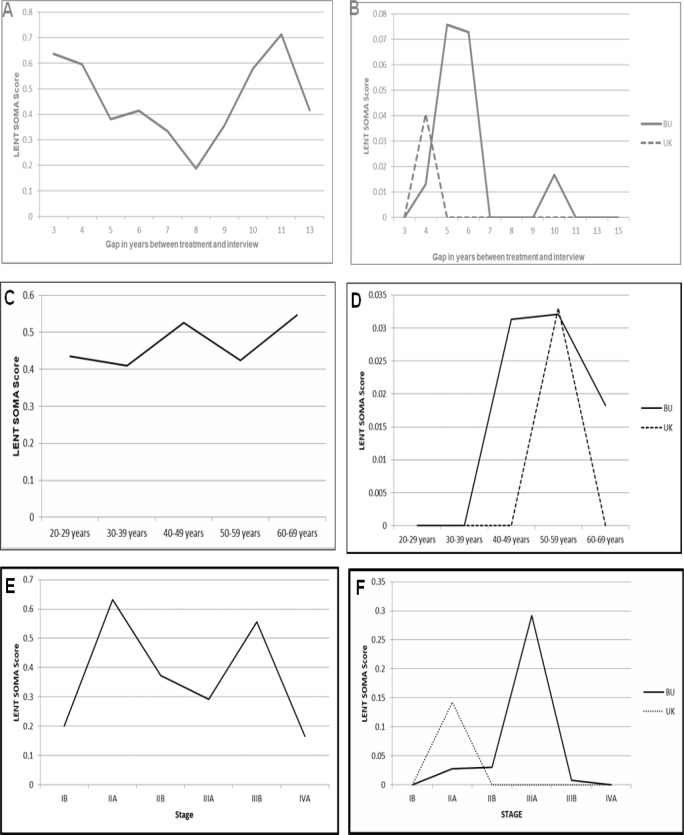
LENT SOMA Scores. (A), Subjective scores according to gap in treatment and interview; (B), Objective scores according to gap in treatment and interview; (C), Age wise distribution of sub- jective scores; (D), Age wise distribution of objective scores; (E), Stage wise distribution of subjective scores; (F), Stage wise distribution of objective scores

**Figure 2:**
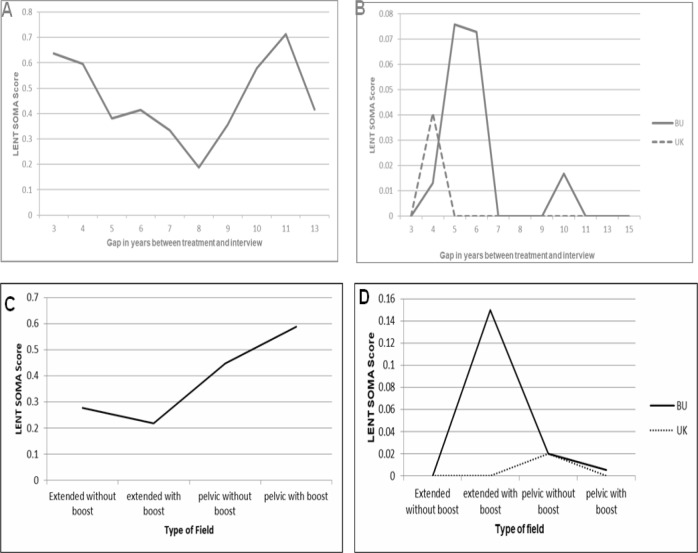
LENT SOMA Scores. (A), Subjective scores in various treatment modalities used alone or in combination; (B), Objective scores in various treatment modalities used alone or in combination; (C), Sub- jective scores in various field types; (D), Objective scores in various field types

**Table 1:** Frequency Distribution of Patients According to Bladder Symptoms after Treatment

Symptoms	No. of patients (For every symptoms n=85)	Percentage
Get up in night to pass urine	72	84.7
Tiredness & Headache together	61	71.8
Urgency in urine	25	29.5
Urine flow weaker than before	14	16.5
Passing less urine	12	14.1
Pain on passing urine	7	8.3
Blood in urine	4	4.8
Ankle swollen	3	3.5
Incontinence of urine	1	1.2
Bladder/Urethra		
Hematuria	2	2.4
Dysuria	2	2.4
Increased frequency urine	1	1.2
Ureter/Kidney		
U/L obstruction	1	1.2

Among the studied patients, 23.5 % were aged less than 40 years, 28.2% fell in age group 40-49, 30.6% in age group 50-59 and 17.64% in age group 60-69 years. Thus majority of patients were noted in age group 40-59 years accounting for 58.81%. 

In this study we found, 24.7% of patients gap between treatment and interview was 4 years followed by 5 years in 12.94% of patients. Evaluation was done after 12 years in 4.7% of patients and after 14 years in 2.35% of patients. 

With regard to stage at presentation, 52.9% of patients included were stage IIIB followed by 29.4 % of patients who presented in stage IIB. Stage IB and IIA patients were 7.1% each and 2.4 % were stage IIIA and 1.2% were stage IV A at presentation. Among the 85 patients 21 (24.7%) patients were having associated co morbid conditions. Ten were hypertensive 3.52% diabetic, and 2.35% of patients had prior history of tuberculosis, hypothyroidism and bronchial asthma each and one patient had association with epilepsy and CVA each. Squamous cell carcinoma was seen in out of 92.94 % of patients. Moderately differentiated SCC was seen in 21.2 % of patients. 3.6 % had poorly differentiated SCC, 4.7 % were SCC with large cell and in 63.5% patients, differentiation was not known. 

In our study, EBRT with concurrent chemotherapy followed by Intracavitary radiotherapy was received by 52.94% patients. Radiotherapy (EBRT+ICR) alone was given in 24.70% of patients. Surgery had been done in 22.34% patients of which twelve patients underwent adjuvant radiation and seven patients received chemo radiotherapy. 

43.52% patients were not treated with chemotherapy. Forty percent of patients received Inj. Cisplatin on Day 1 and 29 and biweekly Inj. 5-FU. Five patients were treated with weekly Inj Paclitaxel and Inj. Cisplatin on Day 1 and 29 each.

Data related to type of field was obtained for 67 patients. Fifty-nine (88.05%) patients were treated with pelvic field and 8(11.93%) with extended field. In pelvic field, 16 (23.88 %) patients received boost where as in extended field, 5 (7.46%) patients received boost. Sixty-three (94.02%) patients were treated with 2 fields technique and 4 (5.97%) received with 4 fields. In 2 field technique, 31.34% were given boost. 96.49 % patients were locally well. recurrences were reported in 3.51% of patients out of which two and one were having local recurrence and metastasis respectively. In this study, 2.4% of patients had hematuria and dysuria each where frequency of urine and unilateral ureteral obstruction were seen in 1.2% of patient each (Table 1).

*LENT SOMA assessment*

The mean subjective bladder score was highest (0.7137) in patients in which gap was 11 years and maximum score was highest (1.46) in patients who were treated 4 years before interview (Figure 1A). Mean objective score BU (Bladder/Urethra) was highest (0.0758) in patients who were interviewed 5 years after treatment and maximum score was highest (0.75) in same group. Mean score in UK (Ureter/Kidney (0.0408) in patients in which gap was 4 years’ maximum score was highest (0.86) in same group. (Figure 1A). When the age wise analysis of scores was done, it was seen that Mean subjective bladder score was seen to be highest in the 60-69 years’ age group (0.5466) and the maximum score was found to be highest (1.46) in the same age group as well (Figure 1C). In the objective scale, Mean score in BU (Bladder/Urethra) was highest in the 50-59 years (0.321) and maximum score was seen to be highest in the 40-49 years’ age (0.75). Mean score in UK (Ureter/Kidney) was highest in the 50-59 years (0.330) and maximum score in this age group was 0.86 (Figure 1D).

In the stage wise analysis, it was seen that, mean subjective bladder score (0.5564) and maximum score (1.46) were highest in stage IIIB patients (Figure 1E). Whereas, mean objective score (0.300) in BU (Bladder/Urethra) and maximum score (0.75) were score were highest in stage IIB and mean score in UK (Ureter/Kidney) was 0.000 in all groups except in stage IIA where it was 0.1429 and maximum score was 0.86 in this group (Figure 1F).

In the analysis of patients according to treatment modalities used, the maximum score in bladder was highest in the Chemo-radiotherapy group (1.46) and lowest in surgery and chemo-radiotherapy (Figure 2A). Whereas in case of mean objective score, BU (Bladder/Urethra) was highest in RT alone group (0.357) and maximum score was highest in Chemo-radiotherapy group (0.75) and in UK (Ureter/Kidney) was highest (0.0408) in RT alone group and zero in all other groups. The maximum score (0.80) was highest in RT alone group (Figure 2B).

In the field wise analysis, the mean subjective bladder score (0.5882) were highest in the pelvic field with boost and maximum bladder score (1.43) were highest in the pelvic field without boost (Figure 2C). On the other hand, we found, Mean objective score BU (Bladder/Urethra) was highest (0.1500) in extended field with boost and maximum score was highest (0.75) in the same group. Mean score in UK (Ureter/Kidney (0.0199) and maximum score (0.86) were highest in the pelvic field without boost group (Figure 2D).

## Discussion

Primary goal of treatment is eradication of tumor. Improvement in treatment modalities results in increased survival rates and with increased survival rates, quality of life after therapy becomes more relevant. For patients with cervical cancer, the objective of new treatment approaches is to improve their survival without compromising Quality of life. In measuring the outcome of treatment, the ability to produce toxicity free survival may be regarded as being as important as achieving disease free survival or local control (Azad and Choudhary, 2010). Urological problems after cervical cancer treatment are a known issue. A study by Azad and Choudhary (2010) titled treatment results of radical radiotherapy of carcinoma uterine cervix using external beam radiotherapy and high dose rate intracavitary radiotherapy on 342 patients reported late complications involving the bowel and bladder in 62 (18.12%) patients treated for cervical cancer by radiotherapy. Bladder complications were seen in 21 (6.14%) patients. Bladder symptoms after radiotherapy are often attributed to a small, contracted and low compliance bladder (International Continence Society, 1981). Radiation toxicity can be divided into acute and late effects. The acute effects of radiotherapy occur during treatment and are self-limiting. In contrast, late effects are manifested several months after treatment and are mediated by damage to the vascular endothelial cells resulting in decreased perfusion and fibrosis (Ralph, 1990). 

If the type of bladder dysfunction is compared, Katepratoom et al., in 2014 found a higher incidence of storage dysfunction (nocturia, urgency , incontinence ) in the concurrent chemo radiotherapy group, although not statistically significant. Parkin et al., in 1999 also found more than half the patients affected by storage problems like nocturia , urgency and urge incontinence compared to voiding difficulty. More studies report coherent results (Manchana et al., 2010; Sedlis et al., 1999). In our study also, more patients complained of nocturia and urgency as compared to voiding difficulties (difficulty in micturition, poor flow).

Majority of patients in our study (57.64%) who had undergone cervical cancer treatment were in age group 40-59 years. In SEER Cancer Statistics Review, 1975-2008, Howlader et al., (2010) showed that from 2004-2008, the median age at diagnosis for cancer of the cervix uteri was 48 years of age. A study done by Routledge et al., (2003) to evaluate the LENT-SOMA scales for prospective assessment of treatment morbidity in cervical carcinoma, the mean age was 54 years (range 24–85 years). Parkin et al., in 1999 reported a mean age 57.0 years (53.6-60.4) in their study. Average objective LENT SOMA scores were seen to be significantly higher in the higher age group patients in our study. Routledge et al., (2003) found no association between patient age and overall LENT subjective score even though bladder/urethra(UC) score decreased with increasing age. 

In our study, both objective and subjective bladder scores were highest 4-5 years after radiotherapy. Marks et al found in their study a mean post treatment interval of 6 years before radiotherapy survivors developed lower urinary tract symptoms (Marks et al., 1995). In a study by Azad and Choudhary (2010), follow up range was 3 to 54 months with a median of 36 months. As observed by Perez and Brady in (1998) and Bomford et al., in 1993, some bladder dysfunction is seen in up to 25% of the patients within 1 to 10 years after radiotherapy However, Pieterse et al., (2006) did not observe deterioration of bladder function in early-stage cervical cancer patients 24 months after treatment.

Davidson et al showed in his study the impact of radiotherapy for carcinoma of the cervix on sexual function assessed using the LENT SOMA scales on 89 patients comprising 28 stage I, 32 stage II, 21 stage III and 7 stage IVA disease (Davidson et al., 2003). In the study conducted by Katepratoom et al. in 2014, 45.7% patients were stage IIIB at presentation. In our study more than half (52.9%) patients presented at advanced stage (stage IIIB). Patients in our set up present at higher stages as compared to the West.

Both average subjective and objective scores were found to be statistically significantly higher in stage III as compared to stage II. This was also seen in the study by Routledge et al., in (2003) where the average overall subjective score rose significantly with advancing stage. It has also been recognized by Pedersen et al., (1994) that advancing stage of disease increases late morbidity after pelvic radiotherapy. 

In our study, EBRT with concurrent chemotherapy followed by Intracavitary radiotherapy was received by 52.94% patients. Radiotherapy (EBRT+ICRT) alone was given in 24.70% of patients. Surgery had been done in 17.64% patients of which 14.11% underwent adjuvant radiation and 4.7 % patients received chemoradiotherapy. In the study done by Routledge et al., (2003), patients were treated with either external beam RT alone (n -7) or external beam RT plus brachytherapy (n -44). Thirty-eight patients received external beam RT with (n 14) or without (n - 24) intracavitary treatment after radical hysterectomy. The combination of surgery and radiotherapy has been confirmed to increase the incidence of lower urinary tract dysfunction when compared with surgery alone or radiotherapy alone. Hazewinkel et al., in (2010) assessed prevalence of bladder symptoms with validated pelvic-floor-related questionnaires and found that surgery combined with radiotherapy caused more urological complications than radiotherapy alone. Lin et al., in (1998) reported abnormal bladder findings in cervical cancer patients treated with surgery plus radiotherapy more than either alone. Their results were statically significant. In our study too, we found higher bladder scores in both radiotherapy alone as well as chemoradiotherapy groups. 

The mean subjective bladder score was highest in the pelvic field with boost and maximum bladder score was highest in the pelvic field without boost. Among objective score, Ureter/Kidney (UK) score was highest in pelvic field without boost. Bladder/Urethra (BU) score was highest in extended field with boost. There is no published data to compare our LENT SOMA score in various treatment fields (Pelvic/Extended with or without boost). 

This is first reported study of India on assessment of Urinary dysfunction in Cervical Cancer patients using LENT SOMA Scales. The aim of this study was to assess the outcome (in terms of LENT SOMA scores) with respect to urological complications in patients of carcinoma uterine cervix treated with multimodality therapy. This study highlights the fact that pelvic radiotherapy causes more urological complications after cervical cancer treatment and difficulties of bladder storage are more common than voiding dysfunction. 

## Conflict of Interest

The author(s) declare no competing financial interests.
